# Assessment of the *N*-Alkylamide Content and Volatile Profiles in Two Cultivars of *Acmella oleracea* (L.) R.K. Jansen Grown in Aquaponics

**DOI:** 10.3390/plants14091401

**Published:** 2025-05-07

**Authors:** Marta Ferrati, Beatrice Bartolini, Giulio Lupidi, Lorenzo Freddi, Valentina Bolletta, Marco Cespi, Rita Giovannetti, Marco Zannotti, Riccardo Petrelli, Filippo Maggi, Eleonora Spinozzi

**Affiliations:** 1 Chemistry Interdisciplinary Project (ChIP), School of Pharmacy, University of Camerino, Via Madonna delle Carceri, 62032 Camerino, Italy; marta.ferrati@unicam.it (M.F.); beatrice.bartolini@studenti.unicam.it (B.B.); giulio.lupidi@unicam.it (G.L.); marco.cespi@unicam.it (M.C.); riccardo.petrelli@unicam.it (R.P.); eleonora.spinozzi@unicam.it (E.S.); 2 MJ Energy srl Società Agricola, Via Lorenzoni, 100, 62100 Macerata, Italy; l.freddi93@gmail.com (L.F.); valebolletta92@gmail.com (V.B.); 3 Chemistry Interdisciplinary Project (ChIP) Research Center, School of Science and Technology, Chemistry Division, University of Camerino, Via Madonna delle Carceri, 62032 Camerino, Italy; rita.giovannetti@unicam.it (R.G.); marco.zannotti@unicam.it (M.Z.)

**Keywords:** aquaponics, jambù, spilanthol, cultivar

## Abstract

*Acmella oleracea* (L.) R.K. Jansen, also called jambù, is a medicinal and aromatic plant native to the Brazilian Amazon rainforest and phytochemically characterized by *N*-alkylamides with spilanthol as the main active compound. Jambù recently attracted the interest of many companies because of its wide range of pharmaceutical, nutraceutical, and cosmetic applications. In this context, it is desirable to identify eco-friendly cultivation methods that not only minimize the environmental footprint but also support the biosynthesis of the plant’s valuable bioactive compounds. The zero-discharge approach of aquaponics makes this growing system an eco-friendly and sustainable production strategy for crops. Thus, a greenhouse experiment was conducted on two jambù cultivars, i.e., cv ‘purple’ and cv ‘yellow’, grown in aquaponic and hydroponic systems. The objective was to compare their contents of *N*-alkylamides, their numbers of capitula, which are the main source of these bioactives, and their volatile profiles. The results highlighted differences between the two cultivars and among plants harvested at different periods. Interestingly, aquaponics yielded plants with a high *N*-alkylamide content, which was comparable to that obtained with hydroponics. Overall, this study highlighted the feasibility of adopting aquaponics to grow *A. oleracea*, paving the way for circular economy-based and sustainable agricultural practices.

## 1. Introduction

Giant strides have been made in the use of sustainable agriculture, which leverages natural resources while avoiding pollutants to maintain environmental equilibrium. In this scenario, aquaponics could promote agroecological sustainability. In fact, this cultivation technique saves up to 90% of water compared with conventional farming methods [1]. Aquaponics is a sustainable and circular-economy-based cultivation system combining the soilless growing of plants (the hydroponic system) with closed-loop aquaculture for farming fish and other aquatic organisms [2]. Being the fusion between hydroponics and aquaculture, aquaponics is attracting interest as a sustainable method for food production [3,4,5]. The transition of nutrients from aquaculture to hydroponics involves several steps. Firstly, water from the aquaculture unit is filtered in a sedimentator to remove suspended solids. Then, it passes through a biofilter, where ammonia, a by-product of fish metabolism, is converted into nitrates. This conversion is facilitated by two classes of aerobic bacteria: nitrifying bacteria (*Nitrosomonas* spp.) oxidize ammonia into nitrites, while nitrosants (*Nitrobacter* spp.) oxidize nitrites into nitrates [6]. Nitrifying bacteria living in the gravel and around plant roots are crucial for nutrient cycling, ensuring the continued functionality of the system [7]. Finally, plants absorb these nitrates through their roots and use them as a nitrogen source, acting as a further filter for the circulating solution [5]. Aquaponics offers the significant advantage of producing two valuable products, i.e., fish and plants, while using the same quantity of water and maximizing the efficient use of non-renewable resources [3]. Additionally, the hydroponic beds act as biofilters, removing ammonia, nitrates, nitrites, and phosphorus from the water [6].

**Figure 1:**
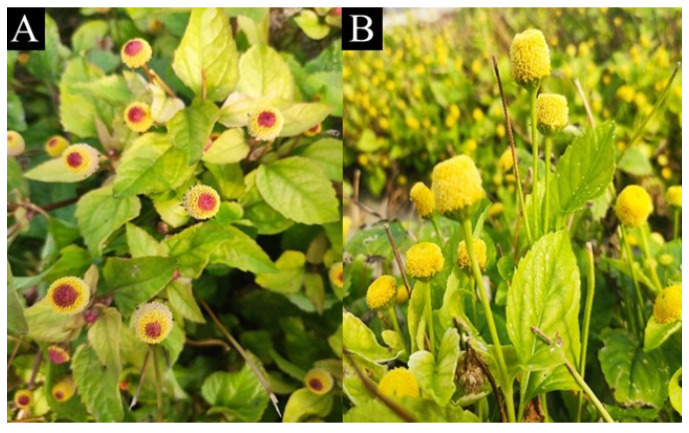
Flowerheads (capitula) of *Acmella oleracea* cv purple (**A**) and cv yellow (**B**).

*Acmella oleracea* (L.) R.K. Jansen (Asteraceae), also known as jambù, is an important medicinal and aromatic plant native to Brazil and valuable for food and beverages (e.g., alcoholic drinks), dentistry, nutraceutical, pharmaceutical, cosmeceutical, and agrochemical industries due to the content of *N*-alkylamides [8,9]. Notably, the extracts and the essential oil (EO) obtained from jambù and spilanthol, i.e., the main *N*-alkylamide, are exploited for their antioxidant, antimicrobial, analgesic, local anesthetic, anti-wrinkle, and insecticidal activities, and several patents have been deposited by cosmetic, food, and pharmaceutical industries [10]. Two cultivars of jambù are distinguished (Figure 1): cv ‘yellow’ features a yellow capitulum-type inflorescence and vigorous light green leaves, while cv ‘purple’ displays capitula with purplish spot, along with intense green leaves that may exhibit purple pigmentation on the foliage and branches [11]. Because of the various applications, finding alternative systems to grow *A. oleracea* cultivars with high *N*-alkylamide content, possibly reducing the environmental footprint, is particularly welcome. Nowadays, its cultivation is carried out mainly in fields and requires significant quantities of soil, water, fertilizers, and pesticides. Thus, it would be desirable to find alternative and sustainable techniques for growing this valuable plant. Moreover, the literature lacks studies comparing the *N*-alkylamide contents of the two different cultivars, which could be helpful in selecting the most suitable for industrial purposes.

In this context, the aim of this work was to evaluate the *N*-alkylamide level, capitula production, and leaf chlorophyll content of the two cultivars of *A. oleracea* grown in aquaponics. Particularly, these responses were compared with those found for the hydroponic system. Given the habitat of *A. oleracea* (wet environments), we hypothesized that aquaponics may fit the ecological needs of this plant. For the sake of completeness, the volatile profile of plants was analyzed using solid-phase microextraction coupled with gas chromatography–mass spectrometry (SPME-GC/MS). All responses were monitored over a period of three months and analyzed using a statistical approach. Moreover, the water quality, which is essential for fish rearing and plant growth, was monitored in both systems during the whole experimental period by measuring pH, electric conductivity (EC), and nutrient content.

## 2. Results and Discussion

### 2.1. Nutrients, pH, and Conductivity of Water

**Figure 2:**
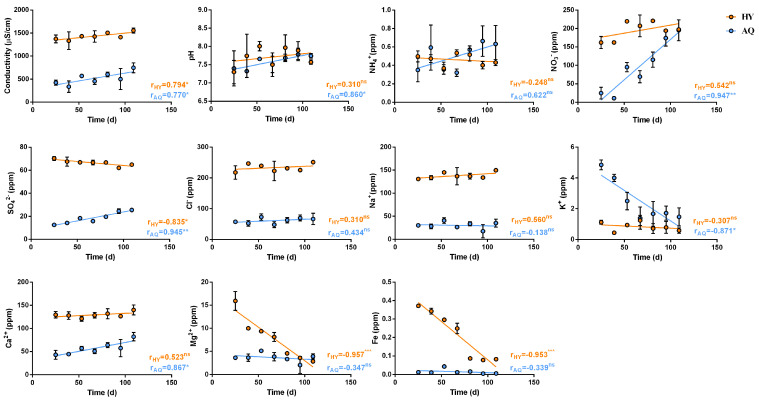
Pearson’s correlation analysis of pH, electrical conductivity (EC), and nutrient contents in the water of aquaponic and hydroponic systems. The r value represents the Pearson coefficient while the apex represents the statistical significance (*p*-value) and has the following means: ^ns^ for *p* > 0.05, * for 0.05 < *p* < 0.01, ** for 0.01 < *p* < 0.001, and *** for *p* < 0.001.

The pH is a factor mainly affecting the access of plants to nutrients. Specifically, the best conditions rely on a slightly acidic pH (6–7), but higher ones (7–8) should still be tolerated [12]. If the pH comes out from this range, plants could suffer a nutrient blockage, becoming unable to use nutrients from water [13]. The pH mainly affects the absorption of Fe, Ca^2+^, and Mg^2+^ [14]. In hydroponics (r = 0.310 ^ns^), there were no significant variations in pH values, while in aquaponics (r = 0.860 *) there was a slight increase over time (Figure 2). Overall, the pH levels of the water in the aquaponic (6.92–7.97) and hydroponic (6.98–8.37) systems were found to be suitable for fish and plant growth and bacteria functionality over the whole experimental period. The electrical conductivity (EC) of water is another crucial parameter in aquaponic and hydroponic systems as nutrient solutions are directly associated with it. Variations in EC may interfere with nutrient availability, stomatal opening, and photosynthetic efficiency, thus affecting plant growth, productivity, and quality [15]. Regarding the trend of conductivity, r = 0.794 * and r = 0.770 * were obtained for hydroponic and aquaponic water, respectively (Figure 2). In both systems, there was a significant increase in salinity over time. Generally, in the hydroponic system, the availability of nutrients usually remains more constant. Concerning aquaponics, the general lower EC (258–824 μs/cm) compared with hydroponics (EC value of 1113–1590 μs/cm) was related to the lower concentration of nutrients. A high EC favors nutrient absorption by plants, whereas a very low EC may cause nutritional deficiencies. However, too high salinity (conductivity greater than 1500 μs) could be harmful to plants [16]. The EC values determined for the aquaponic and hydroponic systems were found to be suitable for plant growth over the whole experimental period. NO_3_^−^ is an important parameter for the aquaponic system, and its accumulation (concentration over 300 mg/L) or depletion usually represents an imbalance between plant requirement and NH_4_^+^ generation [17]. The level of NO_3_^−^ in the hydroponic system remained constant during the experimental period (Figure 2). Indeed, no differences were observed in the values detected over time (r = 0.542 ^ns^). However, the values detected for the aquaponic system showed an increase over time (r = 0.947 **). This could depend on the transformation of feed wastes by bacteria in the tanks. In fact, feed was increased during the experimental period depending on the fish growth. The level of NO_3_^−^, never being higher than 225 ppm, was not toxic to plants [18]. The NH_4_^+^ concentration showed no significant variation over time in both cultivation methods (r = 0.248 ^ns^ and r = −0.622 ^ns^ for hydroponic and aquaponic systems, respectively) (Figure 2). Moreover, the detected levels were not harmful to plant development [19]. Regarding SO_4_^2−^, r = −0.835 * was obtained for the hydroponic system. In fact, a minor decrease in its concentration was observed over time. These nutrients are highly absorbed by plants during the growth phase. In aquaponics, a significant increase in the SO_4_^2−^ concentration over time was observed (r = 0.945 **). This increase is in line with the good functionality of the aquaponic system and denotes the absence of harmful bacteria that could develop under anaerobic or oxygen scarcity conditions. Specifically, they are involved in the reduction of SO_4_^2−^ to sulfur, which is extremely toxic to fish. Sulfur is also involved in the synthesis of proteins essential for plant growth and enzyme and hormone production. No significant changes were observed in the concentration of Cl^−^ and Na^+^ in the water of both crop systems (hydroponics r = 0.310 ^ns^, aquaponics r = 0.434 ^ns^ for Cl^−^; hydroponics r = 0.560 ^ns^, aquaponics r = −0.138 ^ns^ for Na^+^). The higher concentrations of Cl^−^ and Na^+^ in hydroponics could be explained by numerous replenishments of water carried out from the supply network. Regarding K^+^, the concentration did not significantly change in the hydroponic system (r = −0.307 ^ns^) over the experimental period (Figure 2). In the aquaponic system, there was a significant decrease over time (r = −0.871 *). An adequate supply of calcium offers numerous benefits for plants (health of the roots, improvement of water and nutrient absorption in the aquaponic system) and contributes to plant tolerance against unfavorable environmental conditions such as drought and hypersalinity [20]. The concentration of Ca^2+^ in hydroponics did not show a significant change with r = 0.523 ^ns^ and was higher than that in the aquaponic system. However, in aquaponics, there was a significant increase in the concentration of Ca^2+^ over the experiment with r = 0.867 * (Figure 2). The r = −0.957 *** obtained for the Mg^2+^ concentration in the hydroponics revealed a significant decrease over time. This value indicated a constant absorption by plants of this element, which ensured efficient photosynthesis and metabolic efficiency for their growth and development. There were no significant changes in aquaponics (r = −0.347 ^ns^) during the experiment, and the lower concentration did not represent a limit for plant growth in this system. Despite being required in very small quantities, Fe plays a crucial role in the photosynthesis of plants, chlorophyll production, and cellular respiration. Without adequate Fe intake, plants show signs of chlorosis. Fe deficiency is a common problem in aquaponic plants, and it is important to take the necessary measures to integrate chelated Fe into fish feed by foliar application [21]. Specifically, in the aquaponic system, no significant changes or deficiencies in the quantity of Fe were observed (r = −0.339 ^ns^). On the other hand, a meaningful decrease was detected in hydroponics (r = −0.953 ***) (Figure 2).

### 2.2. Capitula Production, N-Alkylamides, and Chlorophyll Content

Given the growing demand for *A. oleracea* derivatives for different industrial applications [22], there is a need for eco-friendly cultivation systems that also preserve the plant’s *N*-alkylamide content. Indeed, the hydroponic system has been developed by some authors [23,24]. However, the cultivation of *A. oleracea* in aquaponics has not been investigated so far. In addition, selecting cultivars with the highest content of these compounds could be desirable. Considering the lack of literature, this experimental work evaluated the phytochemical traits and the capitula production of jambù grown in aquaponics when compared with the hydroponic system. Moreover, the quality of two cultivars, i.e., yellow and purple, was assessed to identify the most appealing one from an industrial perspective. Specifically, the number of capitula (No. capitula/plant), their total *N*-alkylamide content, and the leaf chlorophyll content were monitored. The production of a great number of capitula is an important parameter for the possible exploitation of this plant on an industrial level as this part is the richest source of spilanthol [25]. Since this compound and other *N*-alkylamides are the main compounds responsible for the biological activities of *A. oleracea* [26], their total content in capitula was also considered. Finally, the leaf chlorophyll content was determined to monitor the well-being of plants [27].

**Figure 3:**
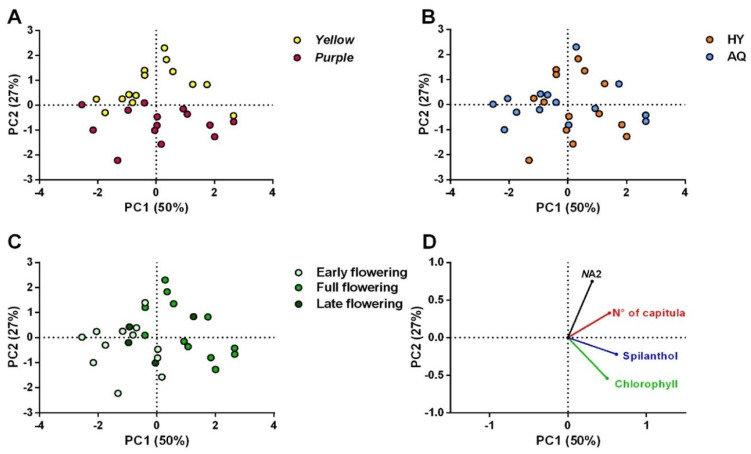
PCA (score plot) of data obtained from HPLC-DAD-MS analysis for *N*-alkylamides and spectrophotometric assay for chlorophyll in plants of yellow and purple cultivars (**A**), grown in aquaponic and hydroponic systems (**B**) and collected in different harvesting periods (**C**). Panel (**D**) represents the loading plot of the PCA.

The above-mentioned responses were evaluated on samples collected every two weeks to also determine the influence of the harvesting period. All the data obtained are shown in Table S1. Their correlation was first determined via Pearson’s analysis (Table S2), showing that (2*Z*)-*N*-isobutyl-2-nonene-6,8-diynamide (*N*A1), (2*E*,7*Z*)-*N*-isobutyl-2,7-decadienamide and (2*E*)-*N*-(2-methylbutyl)-2-undecene-8,10-diynamide (*N*A4/5), and (2*E*,6*Z*,8*E*)-*N*-(2-methylbutyl)-2,6,8 decatrienamide (*N*A6) were strongly correlated with spilanthol. Hence, only spilanthol and (2*E*)-*N*-isobutyl-2-undecene-8,10-diynamide (*N*A2) have been considered and further analyzed in the PCA together with the chlorophyll content and No. capitula/plant. Subsequently, a PCA was drawn up to explore similarities and patterns among the data and to display the influence of the cultivation system, cultivar, and flowering stage on the evaluated responses (Figure 3). As presented in Figure 3A, a clusterization of the data in two groups, i.e., those obtained for cv yellow (upper side) and cv purple (bottom side) emerged. Specifically, capitula and leaves of the latter seemed to present a higher quantity of spilanthol (and correlated compounds) and chlorophyll, respectively, than those of cv yellow. However, a greater content of *N*A2 in the capitula and a higher production of capitula characterized cv yellow. Concerning the influence of the cropping system (Figure 3B), it appeared that aquaponic and hydroponic systems did not show differences, and no clusters were observed. Finally, the PCA highlighted a possible lower concentration of *N*-alkylamides in the capitula and chlorophyll in the leaves collected during early flowering (EF) (Figure 3C). On the other hand, during full flowering (FF) and late flowering (LF), the production of these compounds was higher, and the same trend was observed for the No. capitula/plant. To evaluate the significance of the differences observed among the three harvesting periods and the two cultivars in greater depth, ANOVA followed by the Tukey test or *t*-test was carried out for each evaluated response.

#### 2.2.1. Production of Capitula

**Figure 4:**
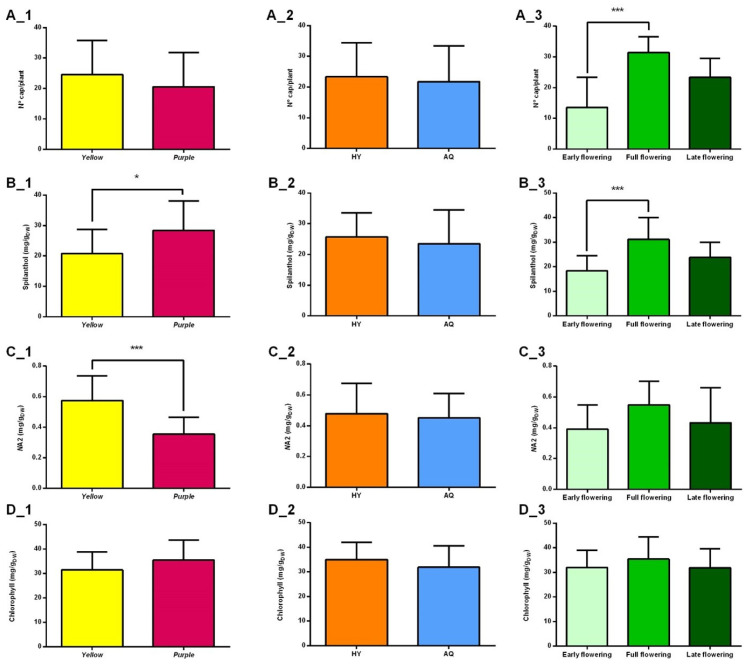
Comparison among average values of production of capitula (**A**), spilanthol (**B**), (2*E*)-*N*-isobutyl-2-undecene-8,10-diynamide (*N*A2) content in capitula (**C**), and chlorophyll content in leaves (**D**). The labels 1, 2, and 3 refer to the comparison among different varieties, cultivation systems, and flowering periods, respectively. The asterisk refers to the results of the *t*-test or ANOVA. The significance was reported in terms of *p*-value as follows: no asterisk for *p* > 0.05, * for 0.05 < *p* < 0.01 and *** for *p* < 0.001.

Even if the PCA apparently showed that cv yellow produced a slightly higher No. capitula/plant with respect to cv purple, the *t*-test did not highlight statistically significant differences (Figure 4(A_1)). These results were not in accordance with those reported by Sousa [28], which showed that cv yellow was extremely more productive with a No. capitula/plant 25.9% higher than cv purple. The diagram shown in Figure 4(A_2) confirmed that the aquaponic and hydroponic systems did not affect the production of capitula. As regards the flowering period, the results of the Tukey test (Figure 4(A_3)) showed that the plants reached their maximum productivity, in terms of capitula, during FF, with a range of 28–44 capitula/plant for cv yellow and approximately 17–36 for cv purple. A statistically significant difference has therefore been observed between the EF and FF periods. Sut et al. [29] also determined the productivity of *A. oleracea* in two different periods, i.e., September and October 2020, finding that there was a greater production of capitula per plant in October than in September.

#### 2.2.2. Spilanthol Content

Figure 4(B_1) shows the results obtained from the *t*-test with the HPLC-determined content of spilanthol in the capitula of cv purple and yellow. The analysis showed statistically significant differences between the two cultivars, with cv purple (14.85–48.0 mg/g) having a higher content of spilanthol than cv yellow (10.93–40.77 mg/g). Interestingly, Figure 4(B_2) suggests that the two cultivation systems did not affect the spilanthol content and the production of capitula. Regarding the harvesting periods, statistically significant differences were found (Figure 4(B_3)). Specifically, the spilanthol content was at the minimum level in capitula collected during EF, increased during FF, and slightly decreased during LF. Overall, the level of spilanthol found in the capitula (11–48 mg/g) was higher than that reported in the literature for in-field cultivation. For instance, Sut et al. [29] found that *A. oleracea* capitula collected from a cultivation site in Southern Italy exhibited a spilanthol content ranging from 0.92 to 1.45%.

#### 2.2.3. (2*E*)-*N*-Isobutyl-2-undecene-8,10-diynamide (*N*A2) Content

A statistically significant difference in the content of *N*A2 was found between cv purple and yellow (Figure 4(C_1)). Specifically, capitula of the latter displayed a concentration of *N*A2 in the range of 0.34–0.76 mg/g, while those of cv purple showed a content of 0.18–0.61 mg/g. The second diagram (Figure 4(C_2)) reports the comparison between the two cultivation methods. Like spilanthol, the difference in *N*A2 concentration between plants grown in aquaponics and hydroponics was not significant. Finally, according to the results obtained from Tukey tests, the variance observed in the content of *N*A2 in capitula among the three harvesting periods was not statistically significant (Figure 4(C_3)).

#### 2.2.4. Chlorophyll Content

Chlorophyll, a fundamental pigment of the photosynthetic machinery, holds a critical role for plant health, and its variation represents a clear indicator of plant stress [30]. Chlorophyll content alteration can be affected by different factors, such as genotype variations, or the age and arrangement of leaves. Furthermore, abiotic factors such as herbicides, temperature, relative humidity, mineral nutrition, and the quality of light can significantly influence the chlorophyll content [31]. The *t*-test and Tukey test did not reveal a statistically significant difference in the chlorophyll content of leaves collected from two cultivars, grown in the two different systems, during the whole period of harvesting (Figure 4(D_1–D_3)). The range found was 20.2–46.8 mg/g. Thus, these results indicated that plants of the two cultivars, in both systems, and during the whole period of analysis were not in a stressed status. The plant’s well-being is primarily upheld by maintaining optimal cultivation conditions, including appropriate humidity and temperature levels, and ensuring adequate quantities of nutrients and minerals.

### 2.3. Volatile Profile

Capitula of cv yellow and purple (collected every two weeks from 7 July to 29 September) were analyzed through SPME-GC/MS for the volatile profile. The resulting chromatograms encompassed about 250 peaks (Figure S1), whose relative percentages (%) (area of the peak with respect to the total area) have been calculated and used for comparative purposes. The compounds with a relative% >1 were considered for the statistical analyses (Table S3). In detail, sesquiterpenes were the most abundant volatiles in both cultivars, being mainly represented by (*E*)-caryophyllene (15.6–26.5% in cv yellow and 19.3–34% in cv purple) and germacrene D (1.1–8.6% in cv yellow and 5.6–9.9% in cv purple). Other minor sesquiterpenes were *α*-humulene, (*Z*,*E*)-*α*-farnesene, and caryophyllene oxide (Table S3). These compounds defend the plant against herbivores and pathogens, attract beneficial organisms, and participate in plant–plant and plant–microbe interactions [32]. The chromatograms were also characterized by monoterpenes, with *β*-pinene (3.9–8.3% in cv yellow and 0.2–6.1% in cv purple) and myrcene (4.1–7.5% in cv yellow and 0.5–8.1% in cv purple) as the most abundant. *β*-Phellandrene and (*Z*)-*β*-ocimene were also detected (Table S3). These compounds are also included in the plant defense mechanisms, particularly for tissue damage protection [33]. Furthermore, some aliphatic compounds were found, with 1-pentadecene (10–20.7% in cv yellow and 6.8–15.4% in cv purple), (*Z*)-6-pentadecene-1-ol (3.9–9% in cv yellow and 1.2–7.8% in cv purple), (*Z*,*Z*)-1,8,11-heptadecatriene (2.3–4.8% in cv yellow and 1.9–3.5% in cv purple), and 1-heptadecene (1.4–2.5% in cv yellow and 1.3–2.2% in cv purple) as the most representative (Table S3). These serve as signaling molecules to attract pollinators or repel herbivores and play a role in the plant defense mechanisms [34]. As expected, spilanthol was also detected (1.9–6.3% in cv yellow and 2.1–14.3% in cv purple). The volatile profile of *A. oleracea* determined in this study is comparable with those reported in the literature referring to the EO [35,36,37]. For instance, Benelli et al. [35] reported (*E*)-caryophyllene (20.8%), *β*-pinene (17.3%), myrcene (17.1%), and caryophyllene oxide (10.0%) as the main volatile compounds. Interestingly, the EO also contained a small amount (3.9%) of spilanthol. Instead, Jerônimo et al. [37] listed caryophyllene oxide (0.3–2.43%), (*E*)-caryophyllene (6.71–27.10%), myrcene (1.14–25.03%), germacrene D (0.06 to 10.27%), 1-pentadecene (3.43%), and *β*-pinene (4.46–10.04%) as the principal volatiles.

**Figure 5:**
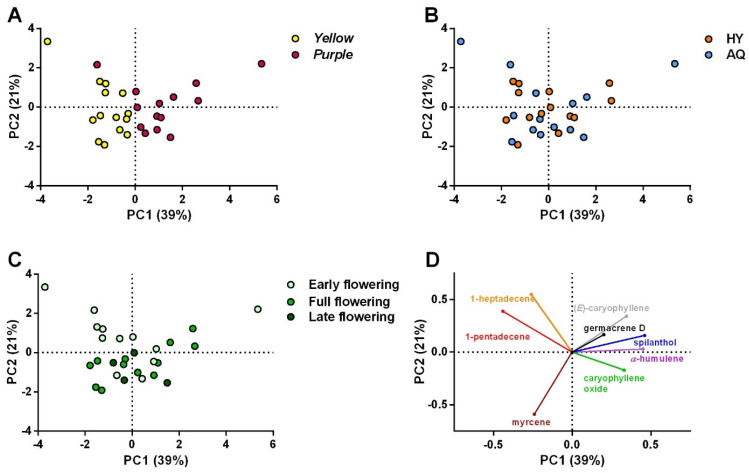
PCA (score plot) of data obtained from SPME-GC/MS analysis of capitula collected from *Acmella oleracea* yellow and purple cultivars (**A**), grown in aquaponic and hydroponic cultivation systems (**B**) and in different harvesting periods (**C**). Panel (**D**) represents the loading plot of the PCA.

The distribution of the detected volatile components was analyzed using a statistical approach to evaluate whether the type of cultivar, cultivation system, and harvesting period influenced the volatile profile of the capitula. The Pearson’s correlation test showed that, in several cases, the relative percentages of components were strongly or very strongly correlated with each other (Table S4). Therefore, only the most relevant components were selected for PCA to avoid redundancy and simplify the interpretation of results. Hence, only myrcene, caryophyllene oxide, 1-heptadecene, (*E*)-caryophyllene, germacrene D, *α*-humulene, 1-pentadecene, and spilanthol were included in the PCA. Overall, the latter was useful to investigate similarities and patterns within the whole dataset. The results observed in the PCA of volatiles mostly overlapped those reported in Section 2.2. In fact, the formation of two clusters was also observed for cv yellow and purple (Figure 5A) in this case. Specifically, cv purple seemed to contain a higher relative % of (*E*)-caryophyllene, germacrene D, spilanthol, *α*-humulene, and caryophyllene oxide. Conversely, the yellow cv exhibited a higher relative abundance of 1-pentadecene, 1-heptadecene, and myrcene. However, the results of PCA indicated no clustering pattern for the two cultivation methods (Figure 5B). This confirmed the results of the PCA performed on *N*-alkylamide, chlorophyll, and No. capitula/plant data. Hence, the two cultivation systems did not show differences from each other in the productivity and chemical profile of the two investigated cultivars. Finally, PCA showed a pattern for the three harvesting periods that did not allow a straightforward interpretation (Figure 5C). It can only be inferred that 1-heptadecene and 1-pentadecene probably reached the highest relative % during the EF period. Conversely, (*E*)-caryophyllene and myrcene were found in higher proportions during FF and LF.

#### Influence of Cultivars, Cultivation Systems, and Harvesting Period

A deeper understanding of the results of PCA was obtained by analyzing the statistical significance of the relative percentage of the compounds as a function of the cultivar type, cultivation system, and harvesting period. Only graphs including statistically significant differences among the samples analyzed are shown in Supplementary Materials.

The *t*-test analysis showed that the two cultivars statistically differed in terms of (*E*)-caryophyllene, spilanthol, *α*-humulene, and 1-pentadecene. Specifically, the capitula of cv purple presented a higher relative percentage of the first three compounds, while a major proportion of 1-pentadecene characterized cv yellow (Figure S2). No statistically significant differences were recorded for all the other components.

The flowering period did not affect the relative percentage of the different volatiles in a statistically significant manner, except for 1-heptadecene, which was found at the highest level during EF (Figure S3). It has been reported that alkenes assist plants by attracting pollinating insects [38,39] and this phenomenon mainly occurs during July and August (corresponding to the EF period). This could explain the higher production of 1-heptadecene during EF (Figure S3).

Finally, no statistically significant differences were found between capitula collected from the aquaponic systems and those from the hydroponic systems. This confirmed that the cultivation technique did not influence the phytochemical traits of *A. oleracea*.

## 3. Materials and Methods

### 3.1. Acmella oleracea Cultivation

The jambù cultivars used in this study were the ‘purple’ or ‘Nazaré’ type and the ‘yellow’ or ‘Jamburana’ type [11]. The seeds of cv purple and yellow were supplied by MJ Energy Srl Società Agricola in Treia (Macerata, Italy). Firstly, they were planted in pots and left to germinate outside the aquaponic and hydroponic cultivation systems. After an incubation period of eight weeks in the nursery, the seedlings were deemed suitable for transplantation. The experiments took place in the greenhouse of MJ Energy Srl Società Agricola in July–September 2023.

#### 3.1.1. Aquaponics

**Figure 6:**
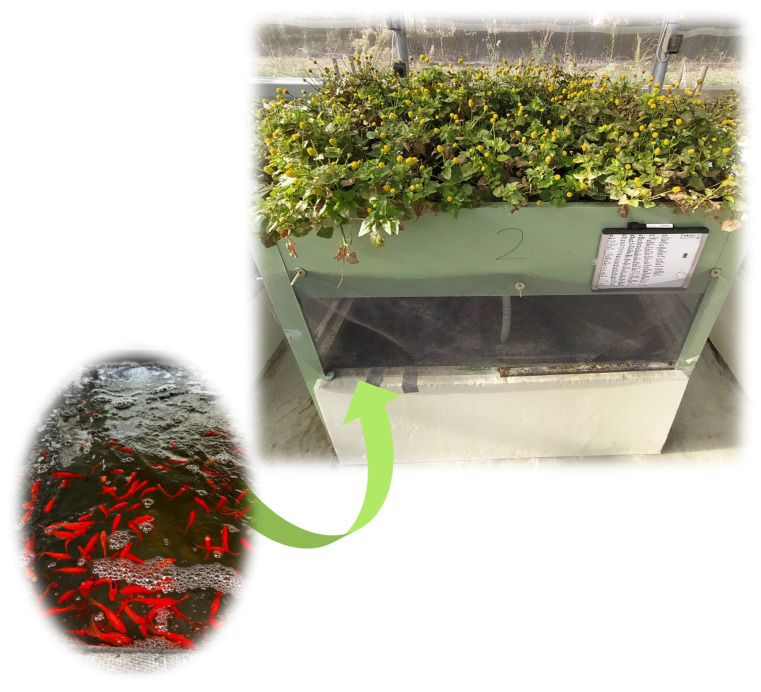
The aquaponic system used for *Acmella oleracea* cultivation.

A total of 1260 juvenile *Carassius auratus* (L.) individuals purchased from Azienda Ittica Frabetti Christian (San Giovanni in Persiceto, Bologna, Italy) were acclimated to the experimental setup for one week in a 500 L tank outfitted with mechanical, biological, and UV filtration systems (MJ Energy Srl Società Agricola). The water parameters were maintained as follows: the temperature was kept at 20.0 ± 0.5 °C, while levels of NH_4_^+^ and NO_2_^−^ were maintained below 0.05 mg/L, and NO_3_^−^ levels were kept below 10 mg/L. Following the acclimation period, the fish, initially weighing 1.2 ± 0.2 g each, were randomly allocated into three Media-Based Aquaponic Systems (Figure 6), each consisting of a 600 L fish tank housing 140 specimens and a 120 L hydroponic unit. The fish were exposed to a natural photoperiod of 14 h of light and 10 h of darkness; the water was maintained at room temperature. A pump (Eheim GmbH & Co., Deizisau, Germany) with a capacity of 1900 L/h regulated the water flow, facilitating 3 water renewals/h. Water from each fish tank was directed to its corresponding hydroponic unit, passing through a siphon fitted with synthetic foam for additional mechanical filtration. In each hydroponic unit, 5 cv purple and 5 cv yellow seedlings were planted, with a density of 3.3 plants/m^2^. Planting was conducted one day before fish were introduced into the aquaponic systems. Each hydroponic unit was furnished with expanded clay to provide physical support for plant growth and mechanical and biological filtration for the fish tanks. The feeding trial spanned 120 days, during which the fish nearly tripled in weight. They were manually fed with experimental diets equivalent to 2% of their body weight, divided into morning and afternoon ratios. Adjustments to the daily feed were made every two weeks based on the weight of a representative sample of fish from each tank. The feed was Veronesi WW2 (Carp Lab, Voghiera, Italy) with a particle size of 2 mm.

#### 3.1.2. Hydroponics

Three systems based on 120 L hydroponic units were used for each experimental group. The tanks were filled with a hydroponic nutrient solution following the Hoagland and Arnon formula [40], and no additional nutrients were introduced during the experiment. A pump (Eheim GmbH & Co., Deizisau, Germany) with a flow rate of 1900 L/h regulated the water flow, facilitating 3 water renewals/h. In each hydroponic unit, 5 cv purple and 5 cv yellow seedlings were planted, with a density of 3.3 plants/m^2^.

### 3.2. Measurements of Water Parameters

The water contained in the aquaponic and hydroponic tanks was analyzed every two weeks until the end of the experiments. The EC and pH of each sample were measured through a HACH Intellical CDC401 and an Intellical PHC101 probe (Hach global, Loveland, CO, USA), respectively. The measurements of nutrients, pH, and water EC were expressed as the means of three independent analyses (each performed in water collected from different tanks).

#### 3.2.1. Ammonium Test

The ammonium test was performed following the APAT 4090 (A2) method [41]. The free and hydrolyzed NH_4_^+^ of the sample reacted with an alkaline solution of potassium iodo-mercurate (Nessler reagent) to form a colored complex according to the following reactions:

- 2(HgI_2_ + KI) + 2NH_3_ → 2(NH_3_HgI_2_) + 2KI;
- 2(NH_3_HgI_2_) → NH_2_Hg_2_I_3_ + NH_4_I.

Then, the absorbance of the colored complex was measured at a wavelength of 420 nm in an Agilent Cary 8454 UV-Vis Diode Array Spectrophotometer (Agilent Technologies, Santa Clara, CA, USA). Specifically, a stock solution (1000 ppm) was prepared and consequently diluted at 0.4, 0.5, 1, 2, and 4 ppm to obtain a calibration curve.

#### 3.2.2. Inductively Coupled Plasma Mass Spectrometry (ICP-MS) Analyses

The water samples were diluted at a ratio of 1:10 in a 1% solution of nitric acid (HNO_3_) and then analyzed using an ICP-MS 7500cx series instrument from Agilent Technologies (Santa Clara, CA, USA). The instrument operated under specific conditions: the power was set at 1550 W, carrier gas flow rate at 0.9 L/min, sample depth at 7 mm, nebulizer pump at 0.1 r.p.s., and spray chamber temperature at 2 °C. Using the collision cell, the 7500cx series instrument can operate in the NoGas/He mode to mitigate most polyatomic interferences. An internal standard solution containing ^45^Sc, ^115^In, ^140^Ce, and ^209^Bi at a concentration of 10 mg/L was prepared from single-element standard solutions (1 g/L, ICP-MS grade, provided by Fluka Analytical, Merck, based in Darmstadt, Germany) and utilized for ICP-MS measurements. Standard solutions of the elements under investigation were prepared by diluting stock solutions (provided by Fluka Analytical, Merck, Darmstadt, Germany) with 1% HNO_3_. The calibration curve for trace elements (Li, Be, B, Al, Ti, V, Cr, Mn, Fe, Co, Cu, Zn, Ga, As, Se, Rb, Sr, Mo, Ru, Pd, Ag, Cd, Sn, Sb, Cs, Ba, Pb, U) was established using solutions at concentrations of 0.01, 0.10, 1.00, 5.00, 10.0, 50.0, 100.0, and 500.0 ppb. For the major elements (Na, Mg, P, S, K, Ca), the calibration curve was prepared using solutions at concentrations of 0.50, 1.00, 2.50, 5.00, 10.0, 25.0, and 50.0 ppm. All calibration standard solutions were prepared using ICP-MS calibration standards (10 mg/L, supplied by Agilent Technologies, Santa Clara, CA, USA).

#### 3.2.3. Ion Chromatography

The anionic composition of the water samples was analyzed using ion chromatography (IC) with a Dionex ICS-1000 apparatus (Sunnyvale, CA, USA). The setup included a Dionex Reagent-free Controller 30 (RFC 30) for gradient elution, an AS50 auto sampler, a conductimetric detector, a Dionex AERS 500 4 mm ionic suppressor, and Chromeleon software (Thermo Scientific, v.6.80) for data acquisition. IC separations were conducted using a Dionex IonPac^®^ AG24A (4 × 50 mm) precolumn and a Dionex IonPac^®^ AS19 (4 × 250 mm) column. The mobile phase consisted of KOH at a 1.0 mL/min flow rate, employing a gradient IC method. Initially, the mobile phase composition was 10 mM and remained constant for 10 min. Subsequently, a linear gradient was applied, increasing the KOH concentration to 45 mM over 20 min, followed by a return to the initial conditions within 2 min. The injection volume was 25 µL. Analytes were identified by comparing their retention times with those of a standard mixture for IC (1000 mg/L, TraceCERT, Merck, Darmstadt, Germany). The monitored anions included F^−^, Cl^−^, SO_4_^2−^, NO_3_^−^, NO_2_^−^, Br^−^, and PO_4_^3−^. For quantification, a calibration curve was constructed for each analyte (Table S5).

### 3.3. Plant Collection and Labeling

The leaves and capitula of *A. oleracea* (cv yellow and cv purple) were separately harvested from the two cultivation systems every two weeks over a total period of three months, from 7 July 2023 to 29 September 2023. Table 1 reports the code of each group, depending on the cv, cultivation system, and harvesting date of the sample. For the elaboration of results, samples collected in specific periods were grouped as follows: EF, from 7 July to 4 August; FF, from 1 to 15 September; LF, 29 September.

### 3.4. HPLC-DAD-MS Analysis

#### 3.4.1. Preparation of Samples and Standard Solutions

Stock solutions of spilanthol (AdipoGen AG, Füllinsdorf, Switzerland) were prepared at concentrations of 1000 and 100 ppm in HPLC-grade methanol and stored at −20 °C until chemical analysis. Then, they were diluted to 500, 250, 50, 10, 5, and 1 ppm for the calibration curve. A batch of *A. oleracea* capitula for each cultivation system and cv was dried in a Biosec dryer (Tauro Essiccatori, Camisano Vicentino, Italy) for 24 h. The sample solutions were prepared according to the procedure of Sut et al. [29]. Briefly, *A. oleracea* powder (25 mg) underwent extraction with methanol (15 mL) in continuous sonication for 10 min. The supernatant was collected, and a subsequent extraction was conducted (10 mL of MeOH). The resulting solutions were combined to a final volume of 25 mL, after centrifugation and filtration on a 0.2 μm syringeless filter.

#### 3.4.2. Analytical Conditions

The high-performance liquid chromatography (HPLC) system was an Agilent 1100 series comprising a binary solvent pump, an autosampler, an ion-trap mass (MS) spectrometer, and a photodiode array detector (DAD) controlled by ChemStation (Agilent, v.01.03) and LCMSD (Agilent, v.6.2). The analytical conditions reported by Kavallieratos et al. [42] were adopted to identify and quantify the main *N*-alkylamides in the capitula. Chromatographic separation was conducted on a Luna C18 column (4.6 × 150 mm, i.d., particle size 5 μm) obtained from Phenomenex (Chesire, UK) at 35 °C. The injection volume was 1 μL for the standard solutions and 10 μL for the samples. *N*-Alkylamides were quantified at 220 nm, and the results were expressed as mg/g DW of capitula.

### 3.5. Chlorophyll Measurement

Solutions of 0.1 M ammonium hydroxide (NH_4_OH) and 80% acetone in water were prepared and mixed (ratio 1:9, NH_4_OH/80% acetone solution). The samples (10 mg of leaves) were weighed into an Eppendorf tube to which 1.05 mL of extraction solution was added. A glass pestle and, subsequently, an Ultraturrax (3 min) were used to favor the extraction of chlorophyll; the samples were then placed in the dark at 4 °C for 2 h until complete extraction. At the end of the incubation, Eppendorf tubes were placed in a centrifuge at 9000 rpm, at room temperature, for 5 min. The supernatant of each sample was collected and stored at −10 °C until analysis. For the determination of chlorophyll, 200 μL of the supernatant were diluted with 800 μL of an 80% acetone solution and analyzed in a Varian Cary 1E UV–Visible Spectrophotometer (Palo Alto, CA, USA). All samples analyzed were subjected to a double reading at 645 and 663 nm. The absorption values were used to calculate the total leaf chlorophyll content through the following equations:

Chlorophyll a (mg/mL) = 12.7 A_663_ − 2.69 A_645_ (1)

Chlorophyll b (mg/mL) = 2.69 A_645_ − 4.68 A_663_ (2)

Total Chlorophyll (mg/mL) = Chlorophyll a + Chlorophyll b(3)

The results were expressed as mg/g DW of leaves.

### 3.6. Solid-Phase Microextraction–Gas Chromatography–Mass Spectrometry (SPME-GC/MS)

The capitula volatile profile of the two cultivars grown in aquaponics and hydroponics was studied by SPME in an Agilent 8890 gas chromatograph equipped with a 5977B single quadrupole mass spectrometer (Santa Clara, CA, USA) and an RTC120 PAL autosampler (CTC Analytics AG, Zwingen, Switzerland). Ionization was achieved with an electron ionization (EI) source (70 eV). The temperature of the fiber conditioning station (DVB/C-WR/PDMS) was set at 250 °C, and the fiber was conditioned for 15 min. The sample was prepared by inserting 0.5 g of capitula into a GC/MS vial and incubated for 5 min at a temperature of 100 °C; then, the fiber was exposed to the headspace for 20 min. The desorption time was set at 1 min. The separation of the molecules was achieved using an HP5-MS capillary column (30 m l. × 0.25 mm i.d. × 0.1 μm f.t.), which was thermostated at 50 °C for 5 min and then heated up to 220 °C at 4 °C/min. The temperature was then increased to 280 °C at 11 °C/min and held for 15 min. Finally, it was heated to 300 °C at 15 °C/min and held for 0.5 min. The total running time was approximately 70 min. He was used as a carrier gas at a flow rate of 1 mL/min. The transfer line was set to 280 °C, and the temperature of the ion source and mass analyzer was 230 and 150 °C, respectively. The acquisition was carried out in SCAN mode (29–400 *m*/*z*). The chromatogram was analyzed using MSD ChemStation software (Agilent, version G1701DA D.01.00) and the NIST Mass Spectral Search Program for NIST/EPA/NIH Spectral Library v. 2.3. The identification of volatiles was confirmed by the interactive use of temperature-programmed linear retention indices (RIs) and MS fragmentations with respect to those stored in commercial libraries and a home-made library [43,44,45]. The relative abundance of each component with respect to the total area was obtained through the integration of each peak without using correction factors.

### 3.7. Statistical Analysis

The presence of correlation among different responses was evaluated through Pearson’s correlation test, with a significance level of 5%. When correlation coefficients were found to be statistically significant, the interpretation of their absolute magnitude was reported according to the following cut-off: (absolute value): r > 0.9, very strong correlation; 0.7 < r < 0.89, strong correlation; 0.4 < r < 0.69, moderate correlation; 0.1 < r < 0.39, weak correlation; and r < 0.09, negligible correlation [46]. The comparisons among data obtained for analyzed samples were carried out using a *t*-test or a one-way ANOVA with a significance level of *p* < 0.05. If statistically significant differences were observed in ANOVA, the post hoc Tukey test was applied (a family-wise level of significance of 5% was chosen). In the manuscript and in the figures, the *p*-value is reported as follows: ^ns^ for *p* > 0.05, * for 0.05 < *p* < 0.01, ** for 0.01 < *p* < 0.001, and *** for *p* < 0.001. The Pearson’s correlation test, *t*-test, and ANOVA were performed with Prism v. 6.01 software (GraphPad Inc., Boston, MA, USA). PCA, operating in correlation mode, was performed using Minitab V18.1 (Minitab Inc., San Diego, CA, USA) to preliminarily evaluate the effect of the studied variables on responses. The PCA was applied to display the influence of the cultivation system, cultivar, and flowering stage on the chemical composition and productivity (in terms of the number of capitula produced by plants) of *A. oleracea*.

## 4. Conclusions

*A. oleracea* boasts a multitude of biological activities, making it highly sought after by various industries. Its applications in dental care and cosmetics, owing to its pain-relieving, anti-aging, and skin-firming properties, as well as in food and beverages and nutraceuticals, have fueled a growing demand for this plant. This study aimed to assess the viability of utilizing aquaponics as a sustainable and efficient cropping system, examining key parameters such as capitula production, *N*-alkylamide concentration, volatile emissions in capitula, and leaf chlorophyll content across two cultivars, i.e., yellow and purple. Additionally, the impact of three harvesting periods (EF, FF, and LF) on these responses was investigated. The results revealed significant differences in *N*-alkylamide content between cultivars, with cv purple exhibiting notably higher levels of spilanthol, the primary bioactive compound, compared with cv yellow. Indeed, cv purple could be preferably cultivated if biomass is needed for the extraction of this compound. Consistently, volatile profile analysis also indicated differences between these cultivars, with a significantly higher headspace percentage of spilanthol in cv purple. An influence of the cultivation system on these parameters was not observed, suggesting comparable efficiency between aquaponics and hydroponics. The full flowering period appeared to be the best one for the plant’s industrial exploitation. Overall, the results suggest that aquaponics offers similar efficiency to hydroponics, making it a viable alternative for growing *A. oleracea* with a reduced environmental footprint compared with conventional farming. This study underscores the feasibility and potential benefits of adopting aquaponics for *N*-alkylamides production in *A. oleracea*, paving the way for sustainable agricultural and circular-economy-based practices.

## Figures and Tables

**Table 1:** Labeling of samples depending on the harvesting date, cultivation system, and cultivars.

Code	Cultivar	Cultivation System	Harvesting Date	Flowering Stage
YEAQ7J	yellow	Aquaponic	7 July	EF ^a^
YEHY7J	yellow	Hydroponic	7 July	EF
YEAQ21J	yellow	Aquaponic	21 July	EF
YEHY21J	yellow	Hydroponic	21 July	EF
YEAQ4A	yellow	Aquaponic	4 August	EF
YEHY4A	yellow	Hydroponic	4 August	EF
YEAQ18A	yellow	Aquaponic	18 August	FF ^b^
YEHY18A	yellow	Hydroponic	18 August	FF
YEAQ1S	yellow	Aquaponic	1 September	FF
YEHY1S	yellow	Hydroponic	1 September	FF
YEAQ15S	yellow	Aquaponic	15 September	FF
YEHY15S	yellow	Hydroponic	15 September	FF
YEAQ29S	yellow	Aquaponic	29 September	LF ^c^
YEHY29S	yellow	Hydroponic	29 September	LF
PUAQ7J	purple	Aquaponic	7 July	EF
PUHY7J	purple	Hydroponic	7 July	EF
PUAQ21J	purple	Aquaponic	21 July	EF
PUHY21J	purple	Hydroponic	21 July	EF
PUAQ4A	purple	Aquaponic	4 August	EF
PUHY4A	purple	Hydroponic	4 August	EF
PUAQ18A	purple	Aquaponic	18 August	FF
PUHY18A	purple	Hydroponic	18 August	FF
PUAQ1S	purple	Aquaponic	1 September	FF
PUHY1S	purple	Hydroponic	1 September	FF
PUAQ15S	purple	Aquaponic	15 September	FF
PUHY15S	purple	Hydroponic	15 September	FF
PUAQ29S	purple	Aquaponic	29 September	LF
PUHY29S	purple	Hydroponic	29 September	LF

^a^ EF, early flowering; ^b^ FF, full flowering; ^c^ LF, late flowering.

## Data Availability

The original contributions presented in this study are included in the article and Supplementary Materials. Further inquiries can be directed to the corresponding author.

## Supplementary Materials

The following supporting information can be downloaded at https://www.mdpi.com/article/10.3390/plants14091401/s1: Figure S1: Example of chromatograms obtained from GC-MS analyses of capitula of *Acmella oleracea* yellow and purple cultivars; Figure S2: Comparison among average values, SPME-GC-MS-determined, of (*E*)-caryophyllene, *α*-humulene, 1-pentadecene, and spilanthol of capitula collected from yellow and purple cultivars; Figure S3: Comparison among average values of 1-heptadecene found from SPME-GC-MS analyses of capitula collected during the three flowering periods (early flowering, full flowering, late flowering); Table S1: Responses evaluated in *Acmella oleracea* plantation, including number (No.) of capitula/plant, *N*-alkylamide content in capitula, and chlorophyll content in leaves; Table S2: Results of Pearson’s correlation analysis of the responses determined for each plant, i.e., *N*-alkylamide content, chlorophyll content, and capitula production (No. capitula/plant); Table S3: Average and standard deviation of volatile compounds found in *Acmella oleracea* capitula in three harvesting periods; Table S4: Results of Pearson’s correlation analysis of the volatile compounds found in *Acmella oleracea*; Table S5: Concentration of standard solution used for the calibration curve.

## Abbreviations

The following abbreviations are used in this manuscript:

EC	electrical conductivity
EF	early flowering
FF	full flowering
LF	late flowering
*N*A1	(2*Z*)-*N*-isobutyl-2-nonene-6,8-diynamide
*N*A2	(2*E*)-*N*-isobutyl-2-undecene-8,10-diynamide
*N*A4/5	(2*E*,7*Z*)-*N*-isobutyl-2,7-decadienamide and (2*E*)-*N*-(2-methylbutyl)-2-undecene-8,10-diynamide
*N*A6	(2*E*,6*Z*,8*E*)-*N*-(2-methylbutyl)-2,6,8 decatrienamide
EO	essential oil
