# Supplementary material for: Assessment of the N-Alkylamide Content and Volatile Profiles in Two Cultivars of Acmella oleracea (L.) R.K. Jansen Grown in Aquaponics
Source: Plants (Basel). 2025 May 7;14(9):1401. doi: 10.3390/plants14091401 (PMC12073576; doi:10.3390/plants14091401)
Supplement: Supplementary file 1 [file plants-14-01401-s001.zip › plants-3569075-supplementary.pdf]

Supplementary Material

Assessment of the *N*-Alkylamide Content and Volatile Profiles in Two Cultivars of *Acmella oleracea* (L.) R.K. Jansen Grown in Aquaponics

Marta Ferrati [1], Beatrice Bartolini [1], Giulio Lupidi [1], Lorenzo Freddi [2], Valentina Bolletta [2], Marco Cespi [1], Rita Giovannetti [3], Marco Zannotti [3], Riccardo Petrelli [1], Filippo Maggi [1,*] and Eleonora Spinozzi [1]

[1] Chemistry Interdisciplinary Project (ChIP), School of Pharmacy, University of Camerino, Via Madonna delle Carceri, 62032 Camerino, Italy
[2] MJ Energy srl Società Agricola, Via Lorenzoni, 100, 62100 Macerata, Italy
[3] Chemistry Interdisciplinary Project (ChIP) research center, School of Science and Technology, Chemistry Division, University of Camerino, Via Madonna delle Carceri, 62032 Camerino, Italy
* Correspondence: filippo.maggi@unicam.it

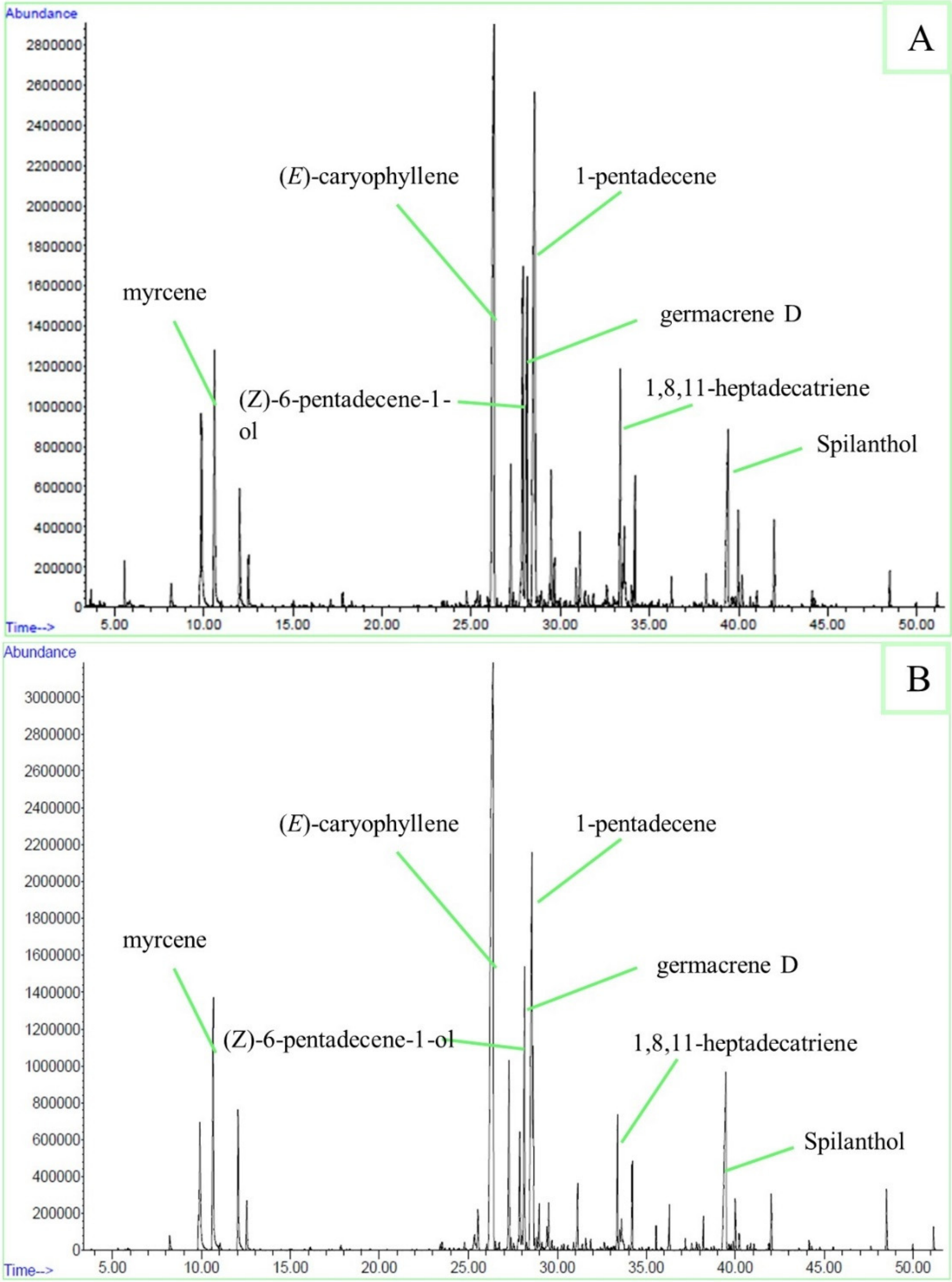

Figure S1. Example of chromatograms obtained from GC-MS analyses of capitula of *Acmella oleracea* yellow (A) and purple (B) cultivars.

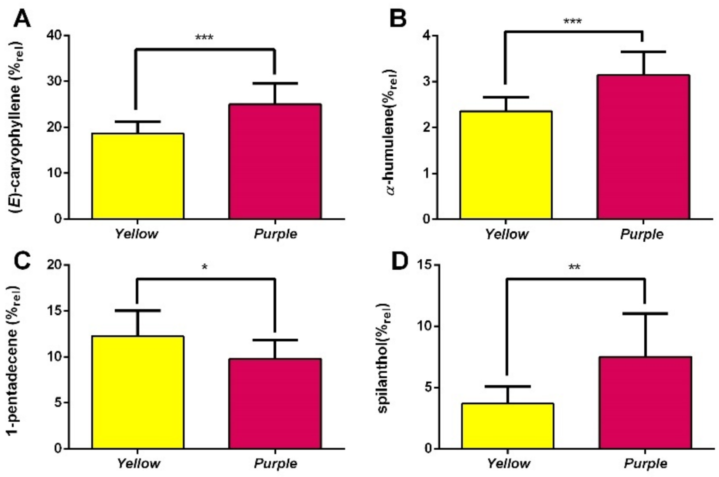

Figure S2. Comparison among average values, SPME-GC-MS-determined, of (*E*)-caryophyllene (A), α-humulene (B), 1-pentadecene (C), and spilanthol (D) of capitula collected from yellow and purple cultivars. The asterisk referees to the results of t-test. The significance was reported in terms of *p*-value as follows: * for $0.05 < p < 0.01$, ** for $0.01 < p < 0.001$, and *** for $p < 0.001$.

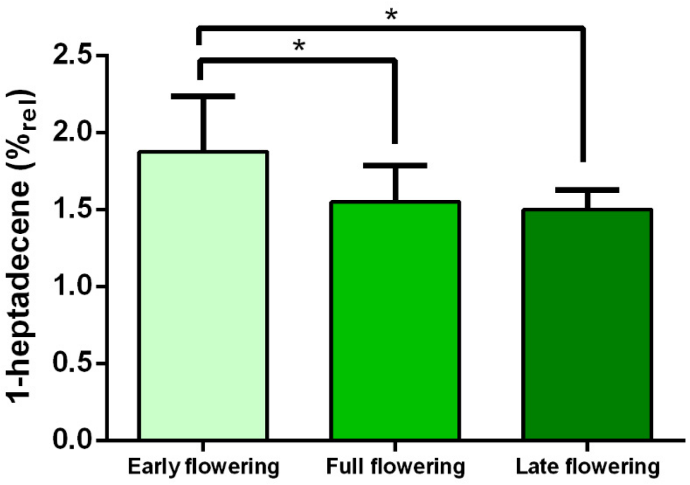

Figure S3. Comparison among average values of 1-heptadecene found from SPME-GC-MS analyses of capitula collected during the three flowering periods (early flowering, full flowering, late flowering). The asterisk referees to the results of ANOVA followed by Tukey test. The significance was reported in terms of *p*-value as follows: * for $0.05 < p < 0.01$.

Table S1. Responses evaluated in *Acmella oleracea* plantation, including number (no) of capitula/plant, *N*-alkylamides content in capitula, and chlorophyll content in leaves.

CODE	No capitula/plant	*NA*1[a] (mg/g DW ± SD[e])	*NA*2[b] (mg/g DW ± SD[e])	Spilanthol (mg/g DW ± SD[e])	*NA*4/5[c] (mg/g DW ± SD[e])	*NA*6[d] (mg/g DW ± SD[e])	Chlorophyll (mg/g DW ± SD[f])
**YEAQ7J**	4.7±06	0.3 ±0.1	0.4±0.1	11.6±1.0	0.1±0.0	0.5±0.0	33.1±2.4
**YEHY7J**	3.7±0.6	0.5±0.0	0.6±0.0	20.0±0.7	0.2±0.0	1.1±0.1	35.0±0.4
**YEAQ21J**	11.7±1.5	0.3±0.1	0.4±0.1	10.9±1.6	0.1±0.0	0.5±0.1	25.1±4.5
**YEHY21J**	15.0±2.6	0.6±0.0	0.7±0.0	23.6±1.6	0.2±0.0	1.6±0.0	26.4±5.1
**YEAQ4A**	27.3±2.5	0.3±0.0	0.4±0.0	14.8±2.3	0.1±0.0	0.9±0.1	30.2±0.3
**YEHY4A**	26.7±0.6	0.3±0.0	0.3±0.1	12.6±1.7	0.2±0.0	0.8±0.1	27.8±4.5
**YEAQ18A**	33.3±4.7	0.7±0.1	0.8±0.1	24.8±4.8	0.2±0.0	1.5±0.2	21.6±0.2
**YEHY18A**	31.7±2.5	0.6±0.1	0.7±0.1	24.5±4.2	0.2±0.0	1.4±0.2	25.5±0.3
**YEAQ1S**	33.7±3.2	0.7±0.0	0.8±0.0	25.1±0.2	0.3±0.0	1.7±0.0	44.3±6.8
**YEHY1S**	33.3±3.1	0.4±0.0	0.5±0.0	18.4±0.0	0.2±0.0	1.2±0.0	24.6±18.2
**YEAQ15S**	41.0±3.0	0.7±0.0	0.5±0.1	40.8±7.6	0.3±0.0	2.7±0.1	44.3±9.5
**YEHY15S**	28.0±1.7	0.6±0.1	0.7±0.1	23.0±4.1	0.3±0.0	1.1±0.2	32.9±5.0
**YEAQ29S**	26.0±2.6	0.4±0.0	0.4±0.0	14.9±0.2	0.1±0.0	0.9±0.0	27.9±18.6
**YEHY29S**	26.7±1.5	0.8±0.1	0.8±0.1	25.8±2.5	0.2±0.0	1.4±0.1	40.9±20.0
**PUAQ7J**	4.3±0.6	0.5±0.0	0.3±0.0	14.9±1.0	0.1±0.0	0.7±0.1	20.2±4.3
**PUHY7J**	2.7±0.6	0.4±0.0	0.2±0.0	17.8±2.6	0.1±0.0	1.3±0.2	42.3±5.4
**PUAQ21J**	8.3±0.6	0.3±0.0	0.2±0.0	14.9±0.3	0.1±0.0	0.8±0.0	27.7±1.0
**PUHY21J**	8.3±0.6	0.7±0.1	0.4±0.0	31.6±1.7	0.4±0.0	2.3±0.1	42.3±5.1
**PUAQ4A**	22.3±3.1	0.5±0.0	0.4±0.0	22.0±0.8	0.2±0.0	1.5±0.1	39.7±11.0
**PUHY4A**	27.3±2.1	0.5±0.0	0.3±0.0	25.0±1.6	0.2±0.0	1.5±0.0	33.8±4.2
**PUAQ18A**	21.3±3.8	0.6±0.1	0.4±0.1	27.9±4.1	0.2±0.0	1.8±0.3	26.3±1.2
**PUHY18A**	27.3±2.5	1.0±0.1	0.5±0.0	35.3±1.6	0.3±0.0	2.1±0.2	36.3±11.2
**PUAQ1S**	25.3±1.5	1.1±0.1	0.6±0.0	48.0±1.9	0.4±0.0	3.0±0.1	45.5±1.8
**PUHY1S**	34.7±1.5	0.80.0	0.4±0.0	36.7±1.6	0.4±0.0	2.6±0.1	46.8±11.0
**PUAQ15S**	33.3±1.5	0.8±0.1	0.4±0.1	29.0±2.7	0.2±0.0	1.8±0.2	36.6±16.8
**PUHY15S**	34.0±2.0	1.0±0.1	0.4±0.1	40.3±4.7	0.4±0.0	2.6±0.2	40.2±19.1
**PUAQ29S**	14.0±1.0	0.9±0.2	0.3±0.1	29.1±4.2	0.2±0.0	2.1±0.3	23.3±11.3
**PUHY29S**	26.7±1.5	0.7±0.1	0.2±0.0	25.5±2.0	0.2±0.0	1.8±0.2	35.3±24.0

[a](2*Z*)-*N*-isobutyl-2-nonene-6,8-diynamide; [b](2*E*)-*N*-isobutyl-2-undecene-8,10-diynamide; [c](2*E*,7*Z*)-*N*-isobutyl-2,7-decadienamide and (2*E*)-*N*-(2-methylbutyl)-2-undecene-8,10-diynamide; [d](2*E*,6*Z*,8*E*)-*N*-(2-methylbutyl)-2,6,8 decatrienamide; [e]The value refers to capitula dry weight and is the mean of three independent analyses ± standard deviation (SD); [f]The value refers to leaves dry weight and is the mean of three independent analyses ± SD.

Table S2. Results of Pearson's correlation analysis of the responses determined for each plant i.e. *N*-alkylamides content, chlorophyll content, and capitula production (no capitula/plant)

**Pearson**	***NA*1**[a]	***NA*2**[b]	**spilanthol**	***NA*4/5**[c]	***NA*$_6$**[d]	**Clorophyll**	**no capitula/plant**
**NA$_1$**		0.505**	0.928***	0.874***	0.780***	0.188^{ns}	0.568***
**NA$_2$**	0.505**		0.367*	0.457**	0.146^{ns}	-0.091^{ns}	0.540***
**spilanthol**	0.928***	0.367*		0.894***	0.927***	0.292^{ns}	0.564***
**NA$_{4/5}$**	0.874***	0.457**	0.894***		0.814***	0.287^{ns}	0.601***
**NA$_6$**	0.780***	0.146^{ns}	0.927***	0.814***		0.347*	0.462**
**Clorophyll**	0.188^{ns}	-0.091^{ns}	0.292^{ns}	0.287^{ns}	0.347*		-0.028^{ns}
**No capitula/plant**	0.568***	0.540***	0.564***	0.601***	0.462**	-0.028^{ns}	

[a](2*Z*)-*N*-isobutyl-2-nonene-6,8-diynamide; [b](2*E*)-*N*-isobutyl-2-undecene-8,10-diynamide; [c] (2*E*,7*Z*)-*N*-isobutyl-2,7-decadienamide and (2*E*)-*N*-(2-methylbutyl)-2-undecene-8,10-diynamide; [d] (2*E*,6*Z*,8*E*)-*N*-(2-methylbutyl)-2,6,8-decatrienamide. The *p*-value shows significancce of r ($p > 0.05$ ns = not significant; $0.05 < p < 0.01$ *; $0.01 < p < 0.001$ **; $p < 0.001$ ***).

Table S3. Average and standard deviation of volatile compounds, found in *Acmella oleracea* capitula in three harvesting periods.

YELLOW

Component	RI[a]	RIL[b]	AQ7J	HY7J	AQ21J	HY21J	AQ4A	HY4A	AQ18A	HY18A	AQ1S	HY1S	AQ15S	HY15S	AQ29S	HY29S
β-pinene	974	974	6.7 ±0.16	4.7 ±0.07	4.8 ±0.13	5.2 ±0.09	3.9 ±0.75	6.0 ±0.10	7.6 ±0.59	5.9 ±0.75	7.4 ± 0.54	8.3 ± 0.20	6.7 ± 0.45	7.2 ± 1.75	7.1 ± 1.34	5.5 ± 0.57
myrcene	992	988	6.9 ±0.28	5.4±0.58	5.1±0.06	4.1±0.18	4.3±0.73	5.4±0.11	7.5±0.18	5.8±0.49	5.9±1	6.3±0.51	6.5±0.39	6.7±1.77	6.5±0.83	6.3 ± 0.39
β-phellandrene	1027	1025	2.9±0.18	2.2±0.05	2.8±0.34	2.1±0.32	1.6±0.14	2.9±0.04	3.8±0.12	3.1±0.30	2.8±0.88	3.7±0.25	3.5±0.16	3.4±1.10	4.2±0.28	3.0 ±0.35
(*Z*)-*β*-ocimene	1039	1032	0.9±0.09	0.9±0.13	0.7±0.19	0.8±0.12	0.9±0.01	1.5±0.1	1.2±0.01	1.2±0.06	0.9±0.65	1±0.35	0.9±0.15	1±0.50	1.1±0.19	0.8 ± 0.16
(*E*)-caryophyllene	1420	1421	20.3±0.31	26.5±5.85	20.2±0.24	17.6±0.30	19.5±0.52	17.2±0.33	15.6±0.26	17.8±0.01	17±2.90	18.2±0.64	17.6±0.16	18.2±3.20	18.3±1.28	18.1 ± 0.35
α-humulene	1450	1452	2.6±0.09	2±0.04	2.9±0.07	2.6±0.02	1.8±0.04	2.7±0.10	2±0.15	2.3±0.08	2.5±0.19	2.5±0.21	2.4±0.04	2.3±0.23	2.3±0.11	2.3 ± 0.04
(*Z*)-6-pentadecene-1-ol	1470	1480	6±0.04	6.7±0.41	5.8±0.06	8.4±0.76	9±0.52	7.2±0.59	5±0.30	4.6±0.56	5.9±0.57	5.5±0.35	4.3±0.16	4.7±0.71	3.9±0.48	5.5 ± 0.34
germacrene D	1478	1484	5.9±0.60	5.9±0.32	6.4±0.4	5.8±2.31	7.6±0.02	6.9±0.52	6.3±0.20	8.6±1.65	4.1±4.67	1.1±1.32	8.1±0.04	4.5±4.56	7.7±0.11	7.5 ± 0.20
1-pentadecene	1490	1492	12.3±0.75	15.2±0.71	12.7±0.52	11.8±0.69	20.7±0.36	12.1±0.84	10.9±0.82	10.2±1.74	10.9±0.16	10.8±0.05	10±0.23	11.2±1.39	10.2±0.52	12.3 ± 0.73
(*Z,E*)-*α*-farnesene	1494	1493	2±0.23	1.6±0.10	1.8±0.17	1.1±0.06	1.3±0.11	0.9±0.29	1.5±0.33	2.1±0.20	1.4±0.84	1.1±0.13	2.1±0.13	2±1.03	2.3±0.01	2.7 ± 0.12
caryophyllene oxide	1578	1582	1.9±0.09	1.1±0.13	2.2±0.28	1.6±0.26	0.7±0.12	1.7±0.04	1.3±0.13	1.7±0.32	1.8±0.58	1.9±0.28	1.6±0.11	1.6±0.33	2.2±0.47	1.6 ± 0.17
(*Z,Z*)-1,8,11-heptadecatriene	1658	1665	3.2±0.17	3.3±0.47	3.6±0.08	4.0±0.28	4.8±0.61	3.7±0.20	2.4±0.23	3.1±0.02	3.1±0.40	2.4±0.08	2.6±0.02	3.2±0.43	2.3±0.33	2.8 ± 0.15
1-heptadecene	1688	1692	1.4±0.10	1.8±0.06	2.1±0.11	2.2±0.10	2.5±0.07	2.2±0.2	1.4±0.04	1.7±0.12	1.9±0.25	1.4±0.09	1.7±0.07	1.9±0.40	1.4±0.08	1.5 ± 0.01
spilanthol	1888	1888	3.7±0.52	5.2±0.13	2.8±0.18	3.1±1.35	3.2±0.01	2.6±0.13	3.9±0.06	5.6±0.49	2.5±1.93	1.9±1.05	6.3±1.53	2.2±1.11	3.2±1.19	5.4 ± 1.44

PURPLE

Component	RI[a]	RIL[b]	AQ7J	HY7J	AQ21J	HY21J	AQ4A	HY4A	AQ18A	HY18A	AQ1S	HY1S	AQ15S	HY15S	AQ29S	HY29S
β-pinene	974	974	4±0.38	3.3±0.23	0.2±0	3.6±1.21	4.8±0.68	5.1±0.30	4.7±1.99	3.3±0.51	2.8±0.23	2.5±2.03	6.1±0.17	2.5±1.99	4±1.84	4.8±0.92
myrcene	992	988	5.9±0.59	5.6±2.14	0.5±0.01	7.3±2.04	5.1±0.56	8.1±0.29	6±1.18	6.2±0.75	5.2±0.13	4.2±2.98	6.9±1.38	4.5±3.27	6.9±1.81	5.7±2
β-phellandrene	1027	1025	3.4±0.78	2.3±0.54	0.2±0.01	3.7±1.20	2.4±0.46	3.9±0.30	2.8±0.38	3±0.44	2.6±0.23	2±1.45	3.5±0.49	2.1±1.13	3.2±0.92	2.9±1.10
(*Z*)-*β*-ocimene	1039	1032	1±0.17	0.9±0.19	0.1±0	1.3±0.32	1.1±0.16	1.3±0.21	0.7±0.04	1±0.42	0.8±0.23	0.6±0.45	1.1±0.02	0.6±0.59	0.9±0.24	0.9±0.23
(*E*)-caryophyllene	1420	1421	34±0.60	29±9.30	32.9±1.59	26.6±3.54	24.9±1.05	24.3±0.43	20.9±1.20	21.8±3.03	23.9±0.58	26.1±1.73	19.3±1.17	25.1±4.26	19.6±0.15	22±2.06
α-humulene	1450	1452	3.3±0.05	2.8±1	4±0.04	3.9±0.62	2.5±0.10	3.9±0	2.8±0.06	2.9±0.36	3.3±0.25	3.4±0.41	2.5±0.25	3.2±0.23	2.8±0.06	2.8±0.02
(*Z*)-6-pentadecene-1-ol	1470	1480	2.1±0	4.8±3.54	1.2±0.38	3.4±1.48	7.8±0.49	3.3±0.19	3.4±1.15	3.2±1.24	3±0.54	2.3±0.06	3.7±0.78	2.3±0.76	2.5±0.66	4.5±0.34
germacrene D	1478	1484	5.6±0.13	6.5±0.29	6.7±0.37	6.1±0.16	7.4±0.16	6.8±0.22	7.1±3.60	8.4±0.56	8.9±0.13	7.5±0.97	7.9±0.20	9.9±1.68	7.6±0.31	6.9±0.19
1-pentadecene	1490	1492	11.4±0.66	11.1±1.84	6.8±1.35	8.3±2.44	15.4±0.11	9.6±0.28	9.4±1.61	10.3±2.86	9.5±0.81	8.2±1.08	9.3±0.33	9±1.63	8.2±2.03	10.9±1.44
(*Z,E*)-*α*-farnesene	1494	1493	2.7±0.41	2.8±0.66	3.3±0.04	2.1±0.11	1.6±0.16	1.8±0.45	2.4±1	1.5±1.90	2.5±0.08	3.8±0.66	2.6±0.69	3±0.45	3.3±0.28	2.2±0.27
caryophyllene oxide	1578	1582	1.2±0.14	0.9±0.02	3.5±0.04	1.6±0.48	1±0.29	1.5±0.02	1.9±0.66	1.5±0.05	1.3±0.13	1.3±0.08	1.8±0.13	2.1±0.57	2.6±0.89	1.6±0.11
(*Z,Z*)-1,8,11-heptadecatriene	1658	1665	1.9±0.29	3.2±1.97	2.1±0.24	2.5±0.78	3.5±0.49	2.4±0.08	1.9±0.42	2.0±0.38	2.6±0.18	2.5±0.08	2.4±0.69	2.1±0.32	2.2±0.59	2.8±0.02
1-heptadecene	1688	1692	1.3±0.13	1.7±1.00	1.7±0.01	1.9±0.76	2.2±0.17	1.5±0.01	1.2±0.24	1.3±0.42	1.6±0.09	1.7±0.09	1.5±0.45	1.4±0	1.4±0.30	1.7±0.03
spilanthol	1888	1888	5±0.11	5.5±0.24	12.7±0.47	3.8±1.13	3.9±0.21	2.1±0.02	7.2±5.88	9.3±5.25	9.8±1	14.3±11.29	6.8±0.04	10.1±3.67	9.2±5.25	5.7±1.86

[a]RI: Retention Index on HP-5MS calculated using homologous series of C_7-C_{30} alkanes; [b]RI_L: Retention Index from Adams or NIST. Percentage values ± standard deviation (SD) are means of three independent analyses.

Table S4. Results of Pearson's correlation analysis of the volatile compounds found in *Acmella oleracea*.

	β-pinene	myrcene	*β*-phellandrene	(*Z*)-*β* ocimene	(*E*)-caryophyllene	*α*-humulene	(*Z*)-6-pentadecene-1-ol	germacrene D	1-pentadecene	(*Z,E*)-*α*-farnesene	caryophyllene oxide	(*Z,Z*)-1,8,11-heptadecatriene	heptadecene	spilanthol
β-pinene		0.644***	0.701***	0.563**	-0.755***	-0.634***	0.445*	-0.492**	0.197^{ns}	-0.582**	-0.155^{ns}	0.167^{ns}	-0.051^{ns}	-0.733^{ns}
Myrcene	0.644***		0.918***	0.752***	-0.429*	-0.227^{ns}	0.024^{ns}	-0.137^{ns}	-0.036^{ns}	-0.233^{ns}	-0.320^{ns}	-0.191^{ns}	-0.369^{ns}	-0.499**
β-phellandrene	0.701***	0.918***		0.744***	-0.435*	-0.180^{ns}	-0.064^{ns}	-0.186^{ns}	-0.154^{ns}	-0.235^{ns}	-0.172^{ns}	-0.290^{ns}	-0.403*	-0.511**
(*Z*)-*β*-ocimene	0.563**	0.752***	0.744***		-0.369^{ns}	-0.241^{ns}	0.311^{ns}	-0.081^{ns}	0.199^{ns}	-0.531**	-0.473*	0.160^{ns}	0.068^{ns}	-0.633***
(*E*)-caryophyllene	-0.755***	-0.429*	-0.435*	-0.369^{ns}		0.656***	-0.483**	0.091^{ns}	-0.181^{ns}	0.503**	0.023^{ns}	-0.301^{ns}	-0.111^{ns}	0.444*
α-humulene	-0.634***	-0.227^{ns}	-0.1803^{ns}	-0.241^{ns}	0.656***		-0.665***	0.119^{ns}	-0.626***	0.452*	0.363^{ns}	-0.476**	-0.186^{ns}	0.428*
(Z)-6-pentadecene-1-ol	0.445*	0.02^{ns}	-0.064^{ns}	0.311^{ns}	-0.483**	-0.665***		-0.270^{ns}	0.829***	-0.761***	-0.463*	0.885***	0.695***	-0.667***
germacrene D	-0.492**	-0.137^{ns}	-0.186^{ns}	-0.081^{ns}	0.091^{ns}	0.119^{ns}	-0.270^{ns}		-0.108^{ns}	0.4284*	-0.016^{ns}	-0.114^{ns}	-0.079^{ns}	0.538**
1-pentadecene	0.197^{ns}	-0.036^{ns}	-0.154^{ns}	0.199^{ns}	-0.181^{ns}	-0.626***	0.829***	-0.108^{ns}		-0.563**	-0.591***	0.787***	0.587**	-0.508**
(Z,E)-*α*-farnesene	-0.582**	-0.233^{ns}	-0.235^{ns}	-0.531**	0.503**	0.452*	-0.761***	0.427*	-0.563**		0.339^{ns}	-0.550**	-0.425*	0.767***
caryophyllene oxide	-0.155^{ns}	-0.320^{ns}	-0.172^{ns}	-0.473*	0.023^{ns}	0.363^{ns}	-0.463*	-0.016^{ns}	-0.591***	0.339^{ns}		-0.412*	-0.257^{ns}	0.299^{ns}
(Z,Z)-1,8,11-heptadecatriene	0.167^{ns}	-0.191^{ns}	-0.290^{ns}	0.160^{ns}	-0.301^{ns}	-0.476*	0.885***	-0.114^{ns}	0.788***	-0.550**	-0.412*		0.877***	-0.500**
1-heptadecene	-0.051^{ns}	-0.369^{ns}	-0.403*	0.068^{ns}	-0.111^{ns}	-0.186^{ns}	0.695***	-0.079^{ns}	0.587**	-0.425*	-0.257^{ns}	0.876***		-0.329^{ns}
Spilanthol	-0.733***	-0.499**	-0.511**	-0.633***	0.444*	0.428*	-0.667***	0.538**	-0.508**	0.767***	0.299^{ns}	-0.500**	-0.329^{ns}	

The p-value shows the significance of r ($p > 0.05$ ns = not significant; $0.05 < p < 0.01$ *; $0.01 < p < 0.001$ **; $p < 0.001$ ***.

Table S5. Concentration of standard solution used for the calibration curve.

Analyte	*Std 1	Std 2	Std 3	Std 4	Std 5	Std 6	Std 7
**F^-**	0.02	0.06	0.1	0.5	1	2	4
**Cl^-**	0.2	1.2	2	10	20	40	80
**Br^-**	0.02	0.06	0.1	0.5	1	2	4
**NO_3^-**	0.08	0.24	0.4	2	4	8	16
	1	5	10	20	40	60	80
**NO_2^-**	0.02	0.06	0.1	0.5	1	2	4
**SO_4^{2-}**	0.2	1.2	2	10	20	40	80
**PO_4^{3-}**	0.05	0.15	0.25	1.25	2.5	5	10

*Std, Standard.
